# Mechanism-Based Strategies for Prevention of Taxane-Induced Hair Follicle Damage in Cancer Chemotherapy

**DOI:** 10.3390/cancers18091351

**Published:** 2026-04-23

**Authors:** Celina Amaya, Matthew P. Schlumbrecht, Tongyu C. Wikramanayake, Xiang-Xi Xu

**Affiliations:** 1 Department of Radiation Oncology, Miller School of Medicine, University of Miami, Miami, FL 33136, USA; 2 Sylvester Comprehensive Cancer Center, Miller School of Medicine, University of Miami, 1120 NW 14th Street, Miami, FL 33136, USA; 3 Department of Obstetrics, Gynecology and Reproductive Science, Miller School of Medicine, University of Miami, Miami, FL 33136, USA; 4 Dr. Phillip Frost Department of Dermatology and Cutaneous Surgery, Miller School of Medicine, University of Miami, Miami, FL 33136, USA

**Keywords:** taxanes, paclitaxel, chemotherapy, alopecia, prevention, microtubules, cryo-treatment, Tp53, cyclin-dependent kinases, low intensity ultrasound

## Abstract

The taxane family of compounds, including paclitaxel, docetaxel (Taxotere), and cabazitaxel (Jevtana), are common drugs used in chemotherapy for the frontline treatment of most major types of cancer. Alopecia, the dramatic loss of hair, is a common side effect that became a symbol of the suffering of many cancer patients. Concerted efforts have been made to understand the mechanism of taxane toxicity to hair follicles and, thus, prevention methods. Taxanes act by stabilizing cellular microtubules, which consequently cause mitotic arrest and then failure, as microtubules play critical functions in chromosome segregation. Hair follicle matrix cells are highly proliferative and thus are exceedingly sensitive to taxanes. We review the cellular mechanism-based strategies under investigation to counter taxane-induced hair follicle damage. These include the application of cyclin kinase inhibitors to block mitotic entry, the practical method using scalp cooling to reduce exposure of scalp hair follicles to drugs during infusion, the requirement of p53 action for hair follicle damage, and the recently discovered method of using low-intensity ultrasound to break taxane-stabilized microtubules and thus reverse taxane toxicity in hair follicle matrix cells. The concept of low-intensity ultrasound as an antidote to taxanes may have the potential to provide a practical and compelling strategy to counter alopecia in cancer treatment using taxanes. **Tweet:** Taxanes (paclitaxel/docetaxel) are powerful microtubule-stabilizing cancer drugs, but they also cause adverse effects, including alopecia. New research discoveries of temporary microtubule disruption by low-intensity ultrasound may counteract taxane toxicity and prevent alopecia during cancer chemotherapy. “**Mechanistic-based strategies for the prevention of taxane-induced hair follicle damage in cancer chemotherapy**” **OUTLINE:** 1. Taxane/paclitaxel mechanism of action in cancer therapy. 2. Taxane side effects: Alopecia (hair loss). 3. p53 dependence of taxane-induced hair follicle damage. 4. Research efforts to counter taxane -induced alopecia by CDK4/6i. 5. Prevention of taxane chemotherapy side effects using scalp cooling. 6. Discovery of low-intensity ultrasound as an antidote for taxane cytotoxicity, and potential prevention of alopecia in chemotherapy using taxanes. 7. Summary and prospective.

## Simple Summary

Taxanes are highly effective drugs used in combination or alone for the treatment of most major solid tumors, especially metastatic cancer. The major side effects of taxanes are peripheral neuropathy, alopecia, and neutropenia, which are significant burdens for patients and limit the full potential of these anti-cancer drugs. Substantial efforts have been made to address the problem of cytotoxic side effects of taxanes, though strategies remain very limited. Taxanes stabilize and interfere with microtubule function, leading to the ultimate death of cancer cells, but they also kill highly proliferative normal cells, including those in the scalp hair follicle matrix. Currently, scalp cooling is practiced to limit exposure and side effects of the drug during infusion, though the effectiveness is uncertain or limited. It has recently been demonstrated that a brief exposure to low-density ultrasound waves was sufficient to eliminate paclitaxel cytotoxicity to cells by transiently breaking microtubule filaments, which were then relocated to lysosomes for disposal. The discovery and concept of low-intensity ultrasound as an antidote to taxane toxicity provide a practical strategy to counter paclitaxel-induced alopecia during cancer treatment.

## 1. Taxane/Paclitaxel Mechanism of Action in Cancer Therapy

Taxol (paclitaxel) is a key drug in the current treatment of several major solid tumors, including ovarian cancer [1,2]. Taxol/paclitaxel targets beta-tubulins within microtubules, alters microtubule dynamics, and stabilizes the filaments [3,4,5], leading to cell death [6,7]. The major side effects (peripheral neuropathy, myelosuppression, and alopecia) are thought to be caused by the impact of paclitaxel on the dynamics of the microtubules in peripheral neurons and killing of mitotic cells in hair follicles and hematopoietic stem cells in bone marrow [8], including rapidly dividing hair matrix keratinocytes in the hair follicles [9].

Additional investigation suggests that paclitaxel treatment causes the formation of multiple micronuclei (micronucleation) from malignant cancer cells, which have a malleable nuclear envelope [10], and the irreversible rupture of the nuclear envelope causes cell death [11]. This non-apoptotic cell death mechanism may account for the persistent power of the taxane/platinum-based chemotherapy [12].

Recent advances in understanding suggest that, in contrast to targeted therapies, taxanes and platinum agents kill cancer cells by physically rupturing nuclear membranes rather than triggering apoptosis, making their effect independent of the intrinsic programmed cell death mechanism [11]. The non-programmed cell death mechanism possibly accounts for the persistent power of taxane/platinum-based chemotherapy [12].

## 2. Taxane Side Effects: Alopecia (Hair Loss)

Alopecia (hair loss) is a major side effect of taxane-based chemotherapy [13,14,15]. Hair follicles undergo cycles of growth (anagen), regression (catagen), and relative quiescence (telogen) throughout life [16,17]. Normally, 85~90% of scalp hair follicles are in anagen at any given time. During this phase, hair follicle matrix cells undergo rapid proliferation, making them extremely susceptible to antineoplastic agents such as paclitaxel. This results in hair shedding that begins as early as 1–3 weeks after initiation of chemotherapy [18,19,20]. More than 80% of patients receiving paclitaxel develop scalp alopecia, but beard, eyebrows, and eyelashes can also be affected [19]. Hair regrowth usually takes 3~6 months after cessation of chemotherapy [19,20], but in some cases, hair loss is irreversible [19,20].

Among the estimated 1.8 million Americans diagnosed with cancer yearly, alopecia is expected to affect approximately 585,000, or 65% of all patients undergoing chemotherapy. In addition to being one of the most common side effects of cancer therapy, alopecia is also one of the most traumatic and dreaded outcomes experienced by patients, negatively affecting a patient’s perception of appearance, body image, sexuality, and self-esteem. Anticipation of alopecia may even lead to the refusal of life-saving chemotherapy [19,21,22]. Currently, only scalp cooling has been established as a method to limit drug exposure to the scalp and to prevent or reduce alopecia, but its success is limited and unpredictable. Further, scalp cooling caps cannot protect eyebrows, eyelashes, and facial hair. Alopecia is a major, psychologically devastating adverse effect of taxane/paclitaxel chemotherapy, and a symbol of the siege of cancer over patients and society. Therefore, novel approaches to prevent paclitaxel-induced alopecia to improve the quality of life of cancer patients are welcomed.

**Figure 1:**
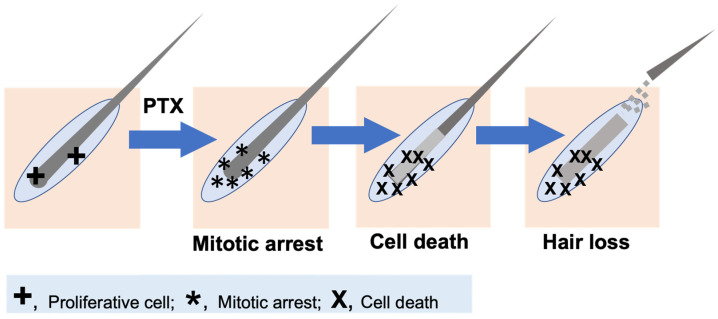
Effects of chemotherapy on the hair matrix cells and mechanism of paclitaxel-induced alopecia. A cartoon illustrates the process of paclitaxel (PTX) causing hair follicle damage and ultimate hair loss. Matrix keratinocytes of hair follicles are proliferative (“+”) and facilitate the growth of the hair shaft. Paclitaxel treatment stabilizes cellular microtubules and interferes with their function at mitotic spindles, causing mitotic arrest (“*”), which leads to an increased presence of monopolar mitotic spindles (“*”). The cells eventually escape mitotic arrest and undergo mitotic catastrophe and cell death (“X”), leading to increased active hair-shaft shedding (exogen).

The underlying pathobiology of taxane chemotherapy-induced alopecia is complex and remains poorly understood [16,20]. In cultured human hair follicles, paclitaxel induces mitotic arrest, massive mitotic defects, and apoptosis in transit-amplifying hair matrix keratinocytes of human scalp hair follicles [9] (Figure 1). The hair matrix keratinocytes proliferate continuously for hair growth, which accounts for the high susceptibility of scalp hair damage from taxanes.

Substantial efforts have been taken to develop strategies to counter chemotherapy-induced alopecia [14,23]. Here, we discuss several strategies that have been considered and developed to counter taxane-induced alopecia. These strategies discussed here are based on clear cellular mechanisms and biological rationales, and are more developed and practical, with a high likelihood for clinical development and commercialization in the coming years. Additional agents that prevent chemotherapy-induced alopecia, such as Minoxidil [24], were developed and investigated. Those agents and methods were developed based on drug screening or testing rather than rational design, and the cellular mechanisms have not yet been understood. These are not included in the current discussion.

## 3. p53 Dependence of Taxane-Induced Hair Follicle Damage

For cancer (for example, ovarian serous carcinomas) treated with taxanes, p53 is not required for chemotherapeutic cancer cell-killing activity, since p53 is mutated and inactivated in the majority of solid tumors treated with taxanes (such as in ovarian cancer).

However, hair follicle damage by paclitaxel may use a different mechanism than that in cancer cells, as studies in mouse models concluded that paclitaxel-induced hair loss may be p53-dependent [18,25,26].

**Figure 2:**
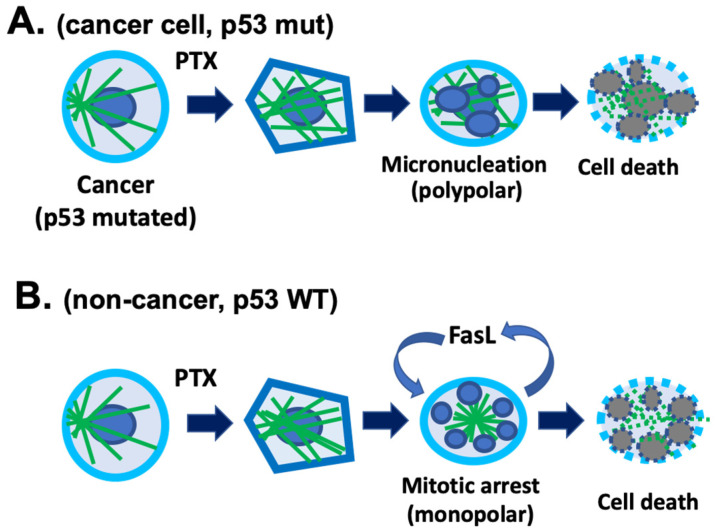
Working model of p53-dependent taxane toxicity in hair matrix cells. (**A**) In cancer cells that harbor p53 mutation (mut), paclitaxel (PTX) induces stabilization and disorganization of cellular microtubules, which subsequently leads to both mitotic-dependent and -independent micronucleation and ultimately cell death. (**B**) In proliferative non-cancer cells, such as hair matrix cells that have wildtype (WT) p53, paclitaxel induces microtubule stabilization and mitotic arrest that is commonly monopolar. Fas receptor and Fas ligand (FasL) autocrine or paracrine signaling is involved in paclitaxel toxicity and cell death in these cells. The hypothesis is that the action of Fas/FasL requires p53 function.

In experiments using a mouse model for chemotherapy-induced hair loss, the results suggest that p53 is essential for the process of chemotherapy-induced hair follicle damage. In contrast to wild-type mice, p53-deficient mice show neither hair loss nor apoptosis in the hair follicle keratinocytes, which maintain active proliferation after cyclophosphamide treatment [25]. Hair follicles in p53 mutants are characterized by down-regulation of Fas and insulin-like growth factor-binding protein 3 and by increased expression of Bcl-2. It was suggested that Fas and c-Kit are involved in chemotherapy-induced cell death in hair follicles [27,28]. The experiments show that Fas is upregulated in hair follicle keratinocytes after cyclophosphamide treatment, and suppression of Fas signaling by either Fas ligand-neutralizing antibody or knockout Fas in mice reduced cyclophosphamide-induced hair follicle involution [27,28]. Thus, the requirement of p53 may be the facilitation of a paracrine or autocrine loop that triggers apoptotic cell death (Figure 2). These observations indicate that local pharmacological inhibition of p53 or the Fas signaling pathway may be useful to prevent chemotherapy-associated hair loss.

Earlier studies reported that paclitaxel upregulates Fas ligand (FasL) to induce apoptosis in cancer cells [29,30,31]. In contrast to the report, additional studies found that anti-FasL antibodies failed to inhibit paclitaxel-induced apoptosis, and neither FasL nor paclitaxel induced apoptosis [32,33]. In consideration of the conflicting results, one possible explanation is that the involvement of Fas–FasL in mediating taxane cytotoxicity is p53-dependent. However, paclitaxel-induced cell death in certain p53-mutated cancer cells is FasL-independent [33], indicating that the involvement of the Fas signaling pathway may be limited to the p53-positive cells (Figure 2). These postulations will need to be further verified and clarified.

In all the cited studies on hair follicle damage found [25,26,27,28], cytotoxic agents such as cyclophosphamide but not taxanes were used to show that p53 is required for chemotherapy drug-induced hair follicle damage. Thus, specific experiments to demonstrate the p53 requirement for taxane-induced hair follicle damage are required.

In sum, these investigations suggest that local suppression of p53 activity, such as applying a p53 inhibitor directly to hair follicles, may have the potential to prevent or reduce chemotherapy-associated hair loss [18]. Additionally, blocking the Fas signaling pathway in hair follicles may also be preventive for chemotherapy-induced alopecia [27,28].

## 4. Research Efforts to Counter Taxane-Induced Alopecia by Inhibition of Cyclin-Dependent Kinases

Since taxanes are considered mitotic inhibitors, blocking cell-cycle progression in cells of the hair follicles may be able to reduce or prevent chemotherapy-induced alopecia. The concept was first tested using cyclin-dependent kinase 2 (CDK2) inhibitor [34,35]. An appealing strategy was the topical application of CDK2 inhibitors to hair-bearing skin to stop the cycling of hair matrix cells when anti-mitotic drugs (such as taxanes) are given systemically to eliminate the tumors. The initial report in mouse models indeed suggested the efficient prevention of hair loss and suppression of hair growth after treatment with chemotherapeutic agents [34]. However, the report was later retracted, and additional effort was not pursued as the reproducibility of the results was not demonstrated [36].

CDK4/6 inhibitors (palbociclib, ribociclib, abemaciclib), which are approved and used to treat breast cancer [37,38,39], were also tested for the potential to prevent taxane side effects. Experiments in mouse models and also in human patients concluded that transient CDK4/6 inhibition protects hematopoietic stem cells from chemotherapy-induced exhaustion [40]. Thus, analogously, CDK4/6 inhibition may be able to stop the cell cycle and protect paclitaxel-induced damage of the proliferative hair follicle matric cells.

In pre-clinical studies using organ cultures of human hair follicles, CDK4/6i was shown to be able to prevent damage to hair follicles by paclitaxel [9]. The experiments were performed in a period of 2 to 5 days, which is a typical time frame suitable for the study of hair follicle organ culture. Further experiments will need to demonstrate if, in longer treatment, and in live animal models (mice), inclusion of CDK4/6i will be able to prevent taxane-induced hair follicle damage and hair loss (or suppression of growth).

However, when used in breast cancer treatment, the drug also causes alopecia, which somewhat reduces the enthusiasm for the development of CDK4/6i as an alopecia preventive drug [37,38,39,41].

## 5. Prevention of Taxane-Induced Alopecia by Scalp Cooling

**Figure 3:**
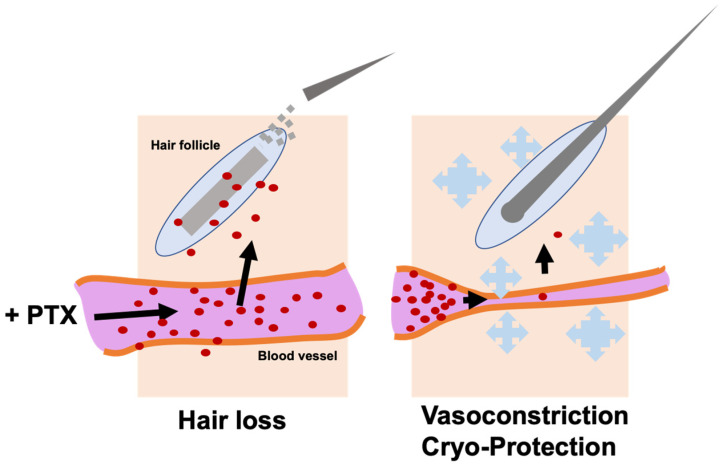
Scalp cooling limits hair follicles’ exposure to paclitaxel through vasoconstriction. Scalp cooling induces local vasoconstriction, reducing blood flow and limiting paclitaxel delivery to hair follicles. Reduced drug exposure prevents hair follicle damage and hair loss (alopecia).

In recent years, scalp cooling systems and cold caps, designed to prevent chemotherapy-induced hair loss, have been used in practice [42,43,44] (Figure 3). Extensive trials of cryo-treatment to prevent alopecia in chemotherapy have been performed, and a large number of reports have been published on the analyses of the effectiveness and benefits [6,44,45,46,47,48,49,50]. Currently, cryo-treatment is practiced to limit exposure and side effects of chemotherapeutic drugs, especially taxanes, during infusion. The rationale is that cooling the scalp during administration of taxanes over several hours reduces blood flow to the site and thus reduces exposure to the drugs [51,52]. Additional biological mechanisms of cooling, such as reduced cellular metabolism, may also help to reduce damage to hair follicles by taxanes [51].

The cooling restricts blood flow and thus exposure of scalp skin and hair follicles to chemotherapeutic agents, though the benefit is often limited or uncertain [23,45,46,47] (Figure 3). Additionally, there is significant patient discomfort associated with scalp cooling, as the taxane infusion can last for three hours or more. Nevertheless, scalp cooling is the best available method currently used in clinical settings for preventing persistent chemotherapy-induced alopecia, albeit with some recognized issues concerning delivery, tolerability, and affordability [46,48]. Not all insurance plans reimburse patients’ costs for FDA-approved cooling systems, and some patients resort to self-administered scalp cooling caps for lower costs and portability, which may be less effective due to user variability [6,53].

Cooling scalp hair follicles to 22 °C or less is required to achieve vasoconstriction of the peripheral blood vessels to reduce drug uptake. To penetrate the 1–2 mm thickness of scalp skin layer, the coolant of 3 to 8 °C was required to achieve a scalp temperature of <22 °C) [54]. Cooling duration is generally set for the entire period of drug administration (3–4 h) plus an hour after. Ideally, an additional 5–6 h of cooling after drug administration should be used to prevent exposure to a substantial drug concentration that remains in circulation. However, this is not done as prolonged cooling greatly increases discomfort.

One study reported that the effectiveness of using a scalp cooling device to protect hair was 61% [42]. However, scalp cooling results in significant side effects and discomfort, such as feeling chilled (which is sometimes unbearable), headache, dizziness, scalp pain, neck pain, etc. [42]. Another report concludes that the DigniCap System is an effective scalp cooling device for the prevention of chemotherapy-induced alopecia in early breast cancer patients. The DigniCap System for scalp cooling was well tolerated and found to be effective in preventing alopecia [55]. It was encouraged to integrate physician and nursing efforts necessary for scalp cooling as a common procedure [56]. However, the experience was not always positive [53], and there is a report stating that “scalp cooling has no place in the prevention of alopecia in adjuvant chemotherapy for breast cancer” [47].

Testing was carried out in vitro using human keratinocyte models to study the effect of cooling on chemotherapy drug-induced cytotoxicity [57]. Cooling mediates protection from chemotherapy-induced cytotoxicity in human keratinocytes by inhibiting cellular drug uptake. The results provide evidence that attenuation of cellular drug uptake represents at least one of the mechanisms underpinning the ability of scalp cooling to prevent alopecia [52]. Laboratory studies in a mouse model have been conducted to verify the rationale of cryo-preservation to prevent hair loss caused by chemotherapy. The results have been positive that cooling reduced paclitaxel-induced hair follicle damage [51]. The mechanisms may include a reduction in blood flow and thus exposure to the paclitaxel agents in the hair follicles [51]. Lower temperature may also slow down the cell cycle, alter metabolism, and have other cellular impacts, but the effects may be limited as the cooling period is only a few hours rather than days.

In sum, scalp cooling is currently the most approved treatment to prevent alopecia due to chemotherapy for cancer. The outcomes are variable, though mostly positive. Laboratory work provides a basis for a mechanism supporting the rationale for use in the prevention of chemotherapy-induced alopecia [14,23,42,46,48] (Figure 3).

## 6. Low-Intensity Ultrasound as an Antidote for Taxane Cytotoxicity and a Potential Strategy for Preventing Chemotherapy-Induced Alopecia

**Figure 4:**
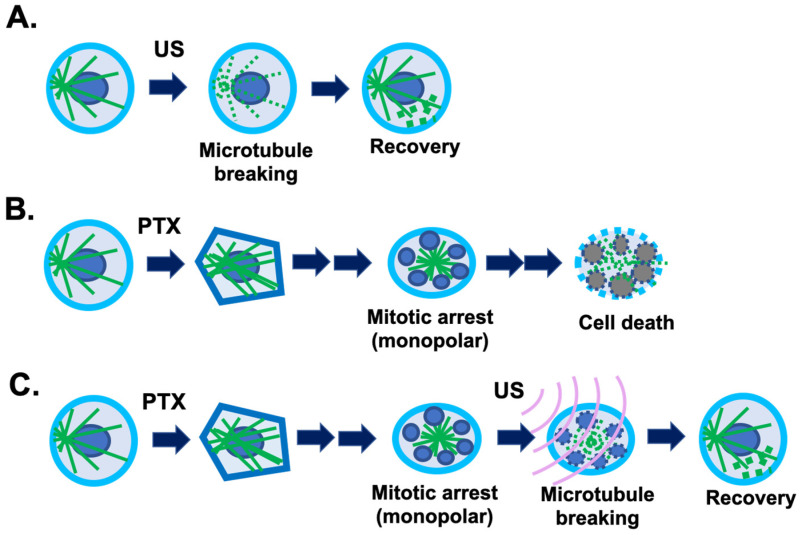
Low-intensity ultrasound working model: Ultrasound exposure reverses cytotoxicity by disrupting rigid microtubule filaments induced by taxane treatment of proliferating cells. Microtubule (MT) bundles are radiated from the organizing center and associate with the nuclear envelope through LINC (linking nuclear and cytoplasmic) complexes. (**A**) Ultrasound (US) is known to transiently disrupt microtubule networks, which reform within 1–2 h. (**B**) Taxol (PTX: paclitaxel, taxanes) induces rigid microtubule filaments that lead to growth arrest, nuclear fragmentation (micronucleation), and subsequent cell death in human scalp hair follicles. (**C**) We suggest a mechanism through which ultrasound reverses cytotoxicity by disrupting rigid microtubule filaments induced by Taxol. The Taxol-bound microtubule fragments are relocated to lysosomes for degradation (illustrated by the bold dots), and new tubulins form a new network of microtubule cytoskeleton without bound Taxol.

A new method under study and development is the use of low-intensity ultrasound to counter taxane cytotoxicity to hair follicles [58]. In a serendipitous laboratory finding, it was reported that briefly (2 to 5 min) exposing proliferative cells in vitro to low-intensity (1 W/cm^2^) ultrasound had little effect on cell proliferation but actually reversed the cytotoxicity of paclitaxel in cultured cells [59]. Ultrasound at low intensity is known to disrupt the microtubule cytoskeleton [60,61] and produce biological effects [62]. Paclitaxel treatment resulted in the appearance of strong staining of microtubule filaments, which was abolished by low-intensity ultrasound [59]. After treatment with ultrasound and recovery, the microtubule cytoskeleton appeared to be the same morphology in paclitaxel-treated cells as in those that did not undergo paclitaxel treatment, but the ultrasound exposure eliminated paclitaxel cytotoxicity [59]. The finding was repeatable in various cell types, and the results conclude that low-intensity ultrasound is capable of eliminating paclitaxel-induced cytotoxicity in all cell types tested by transiently breaking the rigid microtubule filaments [58,59]. A strategy was developed to use this mechanism to counter paclitaxel-induced hair follicle damage and alopecia during chemotherapy using taxanes [58]. The rationale is that ultrasound reverses cytotoxicity by disrupting rigid microtubule filaments induced by taxane treatment of proliferative cells (Figure 4). The physical breakage of paclitaxel-bound microtubules by ultrasound shock waves will result in the relocation of paclitaxel-bound microtubule fragments or tubulin heterodimers to lysosomes for degradation, and new microtubule networks will form rapidly from tubulins not bound to paclitaxel. Tubulin levels in cells are auto-regulated, and newly synthesized tubulins quickly replace degraded paclitaxel-bound tubulins to form the microtubule cytoskeleton [63,64,65]. Thus, a brief pulse exposure to ultrasound efficiently removes the negative impact of paclitaxel on microtubule dynamics and cell cytotoxicity (Figure 4). New preliminary data also suggest that ultrasound dislodges paclitaxel-induced mitotic spindles to avoid mitotic arrest, and thus prevents mitotic catastrophe.

In experiments using human hair follicle organelle culture, a brief exposure to low-intensity ultrasound was able to significantly reduce paclitaxel-induced mitotic arrest and apoptosis of the hair follicle cells [66]. The results conclude that low-intensity ultrasound efficiently and rapidly protects human hair bulbs from paclitaxel-induced cell growth arrest and cell death, presumably by disrupting the drug-induced formation of rigid microtubule bundles and consequently mitotic spindle defects and mitotic catastrophe [66]. The ability of low-intensity ultrasound to prevent paclitaxel-caused hair follicle damage was also demonstrated in live mice [67].

Ultrasound technologies have extensive applications in medicine, either for diagnosis (sonogram) or therapy [68,69,70,71]. Typically, ultrasound with extremely low intensity (1–50 mW/cm^2^) and high frequency (such as 50 MHz) is used for diagnostic (imaging) purposes. High-intensity (>8 W/cm^2^, 20–60 kHz) ultrasound that can deliver strong energy is used for surgery and disruption through heating and acoustic cavitation. The medical application of ultrasound with an intensity that is low yet sufficiently high to produce biological activity is known as ultrasound physiotherapy [69,71], which uses a sufficiently strong but non-disruptive ultrasound shock waves (0.5–3.0 W/cm^2^). The most commonly used devices produce ultrasound waves with frequencies either around 1–3 MHz or 20–150 kHz (known as long wavelength ultrasound). Several studies report similar effects by either 1–3 MHz or 45 kHz ultrasound waves on cells and tissues [72,73,74]. The majority of ultrasound in medical applications of physiotherapy uses frequencies in the range of 1–3 MHz, which traditionally is thought to produce less cavitation and thus less tissue damage. However, more recent laboratory findings indicate that the low-frequency (20 to 100 kHz) ultrasound seems to produce a stronger biological impact [68,69,75,76], and at the same time seems to produce no cell and tissue damage [75,77,78]. With the development of better low-frequency ultrasound devices [79], the use of low-frequency ultrasound for medical procedures has also gained interest [68,69,71].

Ultrasound waves impact cells and may have biological activity even on hair follicles [80]; however, the evidence to support most medical applications of ultrasound is largely insufficient. Most medical applications of ultrasound physiotherapies were determined to lack true merit in large and rigorous clinical studies [70]. Instead, the surprising findings and unique hypothesis promise to introduce the rational, evidence-based use of ultrasound therapy for overcoming paclitaxel cytotoxicity into medical practice [58,59,66].

**Figure 5:**
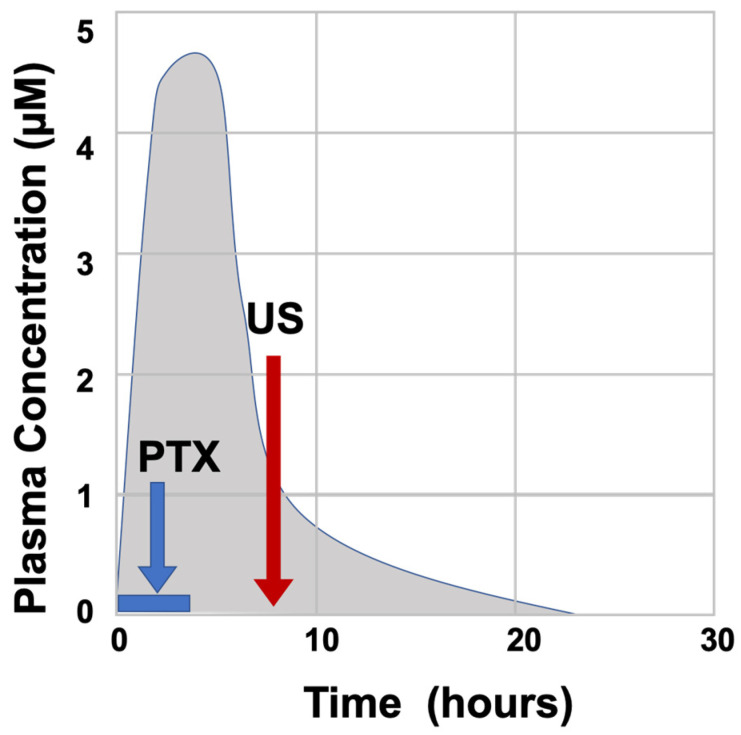
The designed schedule for low-intensity ultrasound treatment in the prevention of alopecia in chemotherapy using paclitaxel/taxanes. Paclitaxel (PTX) is administered by intravenous (IV) route over a period of 3 to 6 h to achieve a plasma peak concentration in 1 to 2 h. Paclitaxel plasma levels fall rapidly following completion of infusion as the drug is rapidly sequestered by binding to cellular microtubules, though presumably drugs taken up by cells will cause cytotoxicity in the next 2–3 days. We reason that ultrasound (US) pulse treatment (5 min exposure) over a period of 4–10 h after drug infusion may be optimal, when plasma drug levels are much lower to prevent further uptake, and the exposure time of scalp hair follicles to the drug remains minimal. Presumably, the local ultrasound treatment will only fragment cellular paclitaxel-bound microtubules in scalp hair follicles but will not interfere with the microtubule stabilization and cytotoxicity of paclitaxel in the tumor cells.

In patients, plasma concentration of paclitaxel decreases rapidly following infusion (Figure 5) [81], although the fraction of drug that enters the tumors will produce cytotoxicity and induce cell death, persisting for several days [5,82]. During the 2–3 days after paclitaxel administration, the drug triggers the death of cancer cells, but also normal cells, including human hair matrix keratinocytes [9]. Thus, for a 3 to 6 h infusion of paclitaxel, it may be suitable to treat patients with low-intensity ultrasound pulse therapy (5 min, about 1 W/cm^2^) over just a few hours (e.g., 4 to 10 h) following chemotherapy. It is known that the human scalp is a strong barrier for the penetration of low-frequency ultrasound energy [71]; thus, the ultrasound intensity will not be able to pass through the scalp bones to the brain area. The additional experiments will need to critically inform the design of a subsequent clinical trial regarding optimal type, dosage, and timing of ultrasound therapy to prevent taxane-induced alopecia.

## 7. Summary and Prospective

Taxane-induced alopecia occurs in up to 80% of patients and is a major impediment to quality of life. Several strategies have been considered and investigated to prevent alopecia or promote hair regrowth [14,23]. Among these, scalp cooling appears to be practical and effective, and is currently used in clinics to prevent alopecia in chemotherapy, though the reported satisfaction and efficacy vary [46,48]. A developing strategy using low-intensity ultrasound to counter taxane activity in scalp hair follicles appears to be another superior strategy [58,59,66], and the ultrasound method requires further investigation to verify effectiveness compared to standard interventions.

Using low-intensity ultrasound to prevent/revert paclitaxel cytotoxicity by disrupting the microtubule cytoskeleton in hair follicle cells to counter paclitaxel-induced alopecia is an innovative concept developed from a serendipitous observation [58,59,66]. As alopecia is such a common and familiar problem in cancer patients, a practical method to prevent hair loss will be a significant symbol of advances in research effort and treatment progress, countering the burden of cancer.

## Data Availability

No new data were created or analyzed in this study. Data sharing is not applicable to this article.

## References

[1] Bookman M.A. (2016). Optimal primary therapy of ovarian cancer. Ann. Oncol..

[2] Runowicz C.D., Wiernik P.H., Einzig A.I., Goldberg G.L., Horwitz S.B. (1993). Taxol in ovarian cancer. Cancer.

[3] Schiff P.B., Fant J., Horwitz S.B. (1979). Promotion of microtubule assembly in vitro by taxol. Nature.

[4] Schiff P.B., Horwitz S.B. (1980). Taxol stabilizes microtubules in mouse fibroblast cells. Proc. Natl. Acad. Sci. USA.

[5] Jordan M.A., Wilson L. (2004). Microtubules as a target for anticancer drugs. Nat. Rev. Cancer.

[6] Weaver D., Pershing M.L., Golden B., Hammel L., Russ P.K., Cripe M. (2024). Retrospective evaluation of Penguin Cold Caps for chemotherapy-induced alopecia. Support. Care Cancer.

[7] Horwitz S.B. (1994). Taxol (paclitaxel): Mechanisms of action. Ann. Oncol..

[8] Canta A., Chiorazzi A., Cavaletti G. (2009). Tubulin: A target for antineoplastic drugs into the cancer cells but also in the peripheral nervous system. Curr. Med. Chem..

[9] Purba T.S., Ng’andu K., Brunken L., Smart E., Mitchell E., Hassan N., O’Brien A., Mellor C., Jackson J., Shahmalak A. (2019). CDK4/6 inhibition mitigates stem cell damage in a novel model for taxane-induced alopecia. EMBO Mol. Med..

[10] Smith E.R., Xu X.X. (2021). Breaking malignant nuclei as a non-mitotic mechanism of taxol/paclitaxel. J. Cancer Biol..

[11] Xu A.P., Xu L.B., Smith E.R., Fleishman J.S., Chen Z.S., Xu X.X. (2024). Cell death in cancer chemotherapy using taxanes. Front. Pharmacol..

[12] Xu L.B., Smith E.R., Koutouratsas V., Chen Z.S., Xu X.X. (2025). The Persistent Power of the Taxane/Platin Chemotherapy. Cancers.

[13] Gaumond S.I., Beraja G.E., Kamholtz I., Ferrari L.M., Mahmoud R.H., Jimenez J.J. (2025). Chemotherapy-Induced Alopecia in Ovarian Cancer: Incidence, Mechanisms, and Impact Across Treatment Regimens. Cancers.

[14] Perez A.M., Haberland N.I., Miteva M., Wikramanayake T.C. (2024). Chemotherapy-Induced Alopecia by Docetaxel: Prevalence, Treatment and Prevention. Curr. Oncol..

[15] Rowinsky E.K., Donehower R.C. (1995). Paclitaxel (taxol). N. Engl. J. Med..

[16] Paus R., Cotsarelis G. (1999). The biology of hair follicles. N. Engl. J. Med..

[17] Shimomura Y., Christiano A.M. (2010). Biology and genetics of hair. Annu. Rev. Genomics Hum. Genet..

[18] Botchkarev V.A. (2003). Molecular mechanisms of chemotherapy-induced hair loss. J. Investig. Dermatol. Symp. Proc..

[19] Trüeb R.M. (2009). Chemotherapy-induced alopecia. Semin. Cutan. Med. Surg..

[20] Paus R., Haslam I.S., Sharov A.A., Botchkarev V.A. (2013). Pathobiology of chemotherapy-induced hair loss. Lancet Oncol..

[21] Chon S.Y., Champion R.W., Geddes E.R., Rashid R.M. (2012). Chemotherapy-induced alopecia. J. Am. Acad. Dermatol..

[22] Rossi A., Fortuna M.C., Caro G., Pranteda G., Garelli V., Pompili U., Carlesimo M. (2017). Chemotherapy-induced alopecia management: Clinical experience and practical advice. J. Cosmet. Dermatol..

[23] Wikramanayake T.C., Haberland N.I., Akhundlu A., Laboy Nieves A., Miteva M. (2023). Prevention and Treatment of Chemotherapy-Induced Alopecia: What Is Available and What Is Coming?. Curr. Oncol..

[24] Chen Y.F., Chen L.H., Yeh Y.M., Wu P.Y., Chen Y.F., Chang L.Y., Chang J.Y., Shen M.R. (2017). Minoxidil is a potential neuroprotective drug for paclitaxel-induced peripheral neuropathy. Sci. Rep..

[25] Botchkarev V.A., Komarova E.A., Siebenhaar F., Botchkareva N.V., Komarov P.G., Maurer M., Gilchrest B.A., Gudkov A.V. (2000). p53 is essential for chemotherapy-induced hair loss. Cancer Res..

[26] Botchkarev V.A., Komarova E.A., Siebenhaar F., Botchkareva N.V., Sharov A.A., Komarov P.G., Maurer M., Gudkov A.V., Gilchrest B.A. (2001). p53 Involvement in the control of murine hair follicle regression. Am. J. Pathol..

[27] Sharov A.A., Li G.Z., Palkina T.N., Sharova T.Y., Gilchrest B.A., Botchkarev V.A. (2003). Fas and c-kit are involved in the control of hair follicle melanocyte apoptosis and migration in chemotherapy-induced hair loss. J. Investig. Dermatol..

[28] Sharov A.A., Siebenhaar F., Sharova T.Y., Botchkareva N.V., Gilchrest B.A., Botchkarev V.A. (2004). Fas signaling is involved in the control of hair follicle response to chemotherapy. Cancer Res..

[29] Srivastava R.K., Sasaki C.Y., Hardwick J.M., Longo D.L. (1999). Bcl-2-mediated drug resistance: Inhibition of apoptosis by blocking nuclear factor of activated T lymphocytes (NFAT) induced Fas ligand transcription. J. Exp. Med..

[30] Biswas R.S., Cha H.J., Hardwick J.M., Srivastava R.K. (2001). Inhibition of drug-induced Fas ligand transcription and apoptosis by Bcl-XL. Mol. Cell. Biochem..

[31] Stumm S., Meyer A., Lindner M., Bastert G., Wallwiener D., Gückel B. (2004). Paclitaxel treatment of breast cancer cell lines modulates Fas/Fas ligand expression and induces apoptosis which can be inhibited through the CD40 receptor. Oncology.

[32] Ferreira C.G., Tolis C., Span S.W., Peters G.J., van Lopik T., Kummer A.J., Pinedo H.M., Giaccone G. (2000). Drug-induced apoptosis in lung cnacer cells is not mediated by the Fas/FasL (CD95/APO1) signaling pathway. Clin. Cancer Res..

[33] Blagosklonny M.V., Robey R., Sheikh M.S., Fojo T. (2002). Paclitaxel-induced FasL-independent apoptosis and slow (non-apoptotic) cell death. Cancer Biol. Ther..

[34] Davis S.T., Benson B.G., Bramson H.N., Chapman D.E., Dickerson S.H., Dold K.M., Eberwein D.J., Edelstein M., Frye S.V., Gampe R.T. (2001). Prevention of chemotherapy-induced alopecia in rats by CDK inhibitors. Science.

[35] Marx J. (2001). Preventing hair loss from chemotherapy. Science.

[36] Davis S.T., Benson B.G., Bramson H.N., Chapman D.E., Dickerson S.H., Dold K.M., Eberwein D.J., Edelstein M., Frye S.V., Gampe R.T. (2002). Prevention of chemotherapy-induced alopecia in rats by CDK inhibitors. Science.

[37] Finn R.S., Crown J.P., Lang I., Boer K., Bondarenko I.M., Kulyk S.O., Ettl J., Patel R., Pinter T., Schmidt M. (2015). The cyclin-dependent kinase 4/6 inhibitor palbociclib in combination with letrozole versus letrozole alone as first-line treatment of oestrogen receptor-positive, HER2-negative, advanced breast cancer (PALOMA-1/TRIO-18): A randomised phase 2 study. Lancet Oncol..

[38] Kwapisz D. (2017). Cyclin-dependent kinase 4/6 inhibitors in breast cancer: Palbociclib, ribociclib, and abemaciclib. Breast Cancer Res. Treat..

[39] Harbeck N., Bartlett M., Spurden D., Hooper B., Zhan L., Rosta E., Cameron C., Mitra D., Zhou A. (2021). CDK4/6 inhibitors in HR+/HER2- advanced/metastatic breast cancer: A systematic literature review of real-world evidence studies. Future Oncol..

[40] He S., Roberts P.J., Sorrentino J.A., Bisi J.E., Storrie-White H., Tiessen R.G., Makhuli K.M., Wargin W.A., Tadema H., van Hoogdalem E.J. (2017). Transient CDK4/6 inhibition protects hematopoietic stem cells from chemotherapy-induced exhaustion. Sci. Transl. Med..

[41] Yang L., Xue J., Yang Z., Wang M., Yang P., Dong Y., He X., Bao G., Peng S. (2021). effects of CDK4/6 inhibitors in the treatment of HR+/HER2- advanced breast cancer: A systematic review and meta-analysis of randomized controlled trials. Ann. Palliat. Med..

[42] Wang S., Yang T., Shen A., Qiang W., Zhao Z., Zhang F. (2021). The scalp cooling therapy for hair loss in breast cancer patients undergoing chemotherapy: A systematic review and meta-analysis. Support. Care Cancer.

[43] Amarillo D., de Boni D., Cuello M. (2022). Chemotherapy, Alopecia, and Scalp Cooling Systems. Actas Dermosifiliogr..

[44] Novice M. (2024). More than just hair. Patient Educ Couns..

[45] Giarratano T., Frezzini S., Zanocco M., Giorgi C.A., Mioranza E., Miglietta F., Griguolo G., Falci C., Faggioni G., Tasca G. (2020). Use of scalp cooling device to prevent alopecia for early breast cancer patients receiving chemotherapy: A prospective study. Breast J..

[46] Rugo H.S., Klein P., Melin S.A., Hurvitz S.A., Melisko M.E., Moore A., Park G., Mitchel J., Bågeman E., D’Agostino R.B. (2017). Association Between Use of a Scalp Cooling Device and Alopecia After Chemotherapy for Breast Cancer. JAMA.

[47] Tollenaar R.A., Liefers G.J., Repelaer van Driel O.J., van de Velde C.J. (1994). Scalp cooling has no place in the prevention of alopecia in adjuvant chemotherapy for breast cancer. Eur. J. Cancer.

[48] Nangia J., Wang T., Osborne C., Niravath P., Otte K., Papish S., Holmes F., Abraham J., Lacouture M., Courtright J. (2017). Effect of a Scalp Cooling Device on Alopecia in Women Undergoing Chemotherapy for Breast Cancer: The SCALP Randomized Clinical Trial. JAMA.

[49] Lambert K.A., Albright B.B., Anastasio M.K., Kaplan S.J., McNally L. (2024). Scalp hypothermia to reduce chemotherapy-induced alopecia: A systematic review and meta-analysis. Gynecol. Oncol..

[50] Contreras M.M., Álvarez B.C., Cavero R.I., Lucerón Lucas-Torres M.I., Jiménez López E., García Maestro A. (2024). Effectiveness of Scalp Cooling to Prevent Chemotherapy-Induced Alopecia in Patients Undergoing Breast Cancer Treatment: A Systematic Review and Meta-analysis. Cancer Nurs..

[51] Chen L., Xu Y., Ye X. (2022). Low temperature mitigating the paclitaxel-induced damages in mouse cell and hair follicle model. Biochem. Biophys. Res. Commun..

[52] Dunnill C., Ibraheem K., Peake M., Ioannou M., Palmer M., Smith A., Collett A., Georgopoulos N.T. (2020). Cooling-mediated protection from chemotherapy drug-induced cytotoxicity in human keratinocytes by inhibition of cellular drug uptake. PLoS ONE.

[53] Kearney C.A., Brinks A.L., Needle C.D., Adhikari S., Marks D.K., Shapiro J., Tattersall I.W., Lo Sicco K.I., Lacouture M.E. (2025). Adverse effects of scalp cooling for the reduction of chemotherapy-induced alopecia: A systematic review and meta-analysis. Breast Cancer Res. Treat..

[54] Ekwall E.M., Nygren L.M., Gustafsson A.O., Sorbe B.G. (2013). Determination of the most effective cooling temperature for the prevention of chemotherapy-induced alopecia. Mol. Clin. Oncol..

[55] Pedersini R., Fornaro C., di Mauro P., Bianchi S., Vassalli L., Amoroso V., Gelmi M., Ardine M., Rodella F., Cosentini D. (2021). Efficacy of the DigniCap System in preventing chemotherapy-induced alopecia in breast cancer patients is not related to patient characteristics or side effects of the device. Int. J. Nurs. Pract..

[56] Peterson L.L., Lustberg M., Tolaney S.M., Ross M., Salehi E., Isakoff S.J. (2020). Integration of Physician and Nursing Professional Efforts to Deliver Supportive Scalp Cooling Care to Oncology Patients at Risk for Alopecia. Oncol. Ther..

[57] Al-Tameemi W., Dunnill C., Hussain O., Komen M.M., van den Hurk C.J., Collett A., Georgopoulos N.T. (2014). Use of in vitro human keratinocyte models to study the effect of cooling on chemotherapy drug-induced cytotoxicity. Toxicol. In Vitro.

[58] Amaya C., Smith E.R., Xu X.X. (2022). Low Intensity Ultrasound as an Antidote to Taxane/Paclitaxel-induced Cytotoxicity. J. Cancer.

[59] Amaya C., Luo S., Baigorri J., Baucells R., Smith E.R., Xu X.X. (2021). Exposure to low intensity ultrasound removes paclitaxel cytotoxicity in breast and ovarian cancer cells. BMC Cancer.

[60] Adler J., Necas O., Hrazdira I. (1993). Disassembly of microtubules due to low intensity ultrasound. Folia Biol..

[61] Samandari M., Abrinia K., Mokhtari-Dizaji M., Tamayol A. (2017). Ultrasound induced strain cytoskeleton rearrangement: An experimental and simulation study. J. Biomech..

[62] Dalecki D. (2004). Mechanical bioeffects of ultrasound. Annu. Rev. Biomed. Eng..

[63] Ben-Ze’ev A., Farmer S.R., Penman S. (1979). Mechanisms of regulating tubulin synthesis in cultured mammalian cells. Cell.

[64] Caron J.M., Jones A.L., Rall L.B., Kirschner M.W. (1985). Autoregulation of tubulin synthesis in enucleated cells. Nature.

[65] Gasic I., Boswell S.A., Mitchison T.J. (2019). Tubulin mRNA stability is sensitive to change in microtubule dynamics caused by multiple physiological and toxic cues. PLoS Biol..

[66] Cheret J., Samra T., Verling S.D., Gherardini J., Rodriguez-Feliz J., Bauman A.J., Sanchez C.A., Wikramanayake T.C., Xu X.X., Paus R. (2023). Low-Intensity Ultrasound as a Potential Intervention Strategy to Protect Human Scalp Hair Follicles from Taxane-Induced Toxicity. J. Investig. Dermatol..

[67] Amaya C., Luo S.H., Smith E.R., Cheret J., Schlumbrecht M.P., Wikramanayake T.C., Paus R., Xu X.X. (2026). Disruption of microtubules with low intensity ultrasound rescues hair follicle damage by paclitaxel. Preprint.

[68] Abramavičius S., Volkevičiūtė A., Tunaitytė A., Venslauskas M., Bubulis A., Bajoriūnas V., Stankevičius E. (2020). Low-Frequency (20 kHz) Ultrasonic Modulation of Drug Action. Ultrasound Med. Biol..

[69] Ahmadi F., McLoughlin I.V., Chauhan S., ter-Haar G. (2012). Bio-effects and safety of low-intensity, low-frequency ultrasonic exposure. Prog. Biophys. Mol. Biol..

[70] Miller D., Smith N., Bailey M., Czarnota G., Hynynen K., Makin I. (2012). Overview of Therapeutic Ultrasound Applications and Safety Considerations. J. Ultrasound Med..

[71] ter Haar G. (2007). Therapeutic applications of ultrasound. Prog. Biophys. Mol. Biol..

[72] Reher P., Doan N., Bradnock B., Meghji S., Harris M. (1998). Therapeutic ultrasound for osteoradionecrosis: An in vitro comparison between 1 MHz and 45 kHz machines. Eur. J. Cancer..

[73] Robertson V.J., Baker K.G. (2001). A Review of Therapeutic Ultrasound: Effectiveness Studies. Physical. Therapy.

[74] Robertson V.J., Ward A.R. (1995). Subaqueous ultrasound: 45kHz and 1MHz machines compared. Arch. Phys. Med. Rehabil..

[75] Iida K., Luo H., Hagisawa K., Akima T., Shah P.K., Naqvi T.Z., Siegel R.J. (2006). Noninvasive low-frequency ultrasound energy causes vasodilation in humans. J. Am. Coll. Cardiol..

[76] Samuels J.A., Weingarten M.S., Margolis D.J., Zubkov L., Sunny Y., Bawiec C.R., Conover D., Lewin P.A. (2013). Low-frequency (<100 kHz), low-intensity (<100 mW/cm(2)) ultrasound to treat venous ulcers: A human study and in vitro experiments. J. Acoust. Soc. Am..

[77] Fischell T.A., Abbas M.A., Grant G.W., Siegel R.J. (1991). Ultrasonic energy. Effects on vascular function and integrity. Circulation.

[78] Scarponi C., Nasorri F., Pavani F., Madonna S., Sestito R., Simonacci M., De Pità O., Cavani A., Albanesi C. (2009). Low-frequency low-intensity ultrasounds do not influence the survival and immune functions of cultured keratinocytes and dendritic cells. J. Biomed. Biotechnol..

[79] Sunny Y., Bawiec C.R., Nguyen A.T., Samuels J.A., Weingarten M.S., Zubkov L.A., Lewin P.A. (2012). Optimization of un-tethered, low voltage, 20–100 kHz flexural transducers for biomedical ultrasonics applications. Ultrasonics.

[80] Liao A.H., Lin K.H., Chuang H.C., Tsai C.H., Lin Y.C., Wang C.H., Shih C.P., Liu H.L. (2020). Low-frequency dual-frequency ultrasound-mediated microbubble cavitation for transdermal minoxidil delivery and hair growth enhancement. Sci. Rep..

[81] Wiernik P.H., Schwartz E.L., Strauman J.J., Dutcher J.P., Lipton R.B., Paietta E. (1987). Phase I clinical and pharmacokinetic study of taxol. Cancer Res..

[82] Michalakis J., Georgatos S.D., de Bree E., Polioudaki H., Romanos J., Georgoulias V., Tsiftsis D.D., Theodoropoulos P.A. (2007). Short-term exposure of cancer cells to micromolar doses of paclitaxel, with or without hyperthermia, induces long-term inhibition of cell proliferation and cell death in vitro. Ann. Surg. Oncol..

